# Supplementary material for: A conceptual and computational framework for modeling the complex, adaptive dynamics of epidemics: The case of the SARS-CoV-2 pandemic in Mexico
Source: PLoS One. 2025 May 8;20(5):e0323473. doi: 10.1371/journal.pone.0323473 (PMC12061415; doi:10.1371/journal.pone.0323473)
Supplement: Attachment — Submitted filename: Response to reviewers.docx [file pone.0323473.s002.docx]

| Comment | Answer or action |
| --- | --- |
| Reviewer 1 |  |
| EPI-Puma integrates data from various sources…” Define the abbreviation “EPI-Puma” when it first appears in the manuscript. The sentence also mentions integrating data from various sources but doesn’t identify which sources are used, based on what justification the data are selected and how they are incorporated. | Strictly speaking, EpI-PUMA is not an abbreviation. The part “EpI” refers to Epidemiological Intelligence. However, the “PUMA” part just refers to the UNAM as the puma is the symbol of the university. Edited to clarify what is EPI-Puma and specify the type of data is identified, collected and then integrated |
| The abstract doesn’t provide how accurate the EPI-puma project and platform are in predicting the outcomes. | The platform allows a user to create an almost unlimited number of models by a choice of class (what to predict), covariables (the predictors) and a time period. Each model has its own classification performance as can be observed in the platform. Thus, how accurate the EpI-PUMA platform is depends on all these choices. This is not a reflection on the Bayesian classifier algorithm used but on the intrinsic predictability of the chosen covariables with respect to the class to be predicted. Thus, a model with just climatic covariables will be seen to be less predictive than a model that uses mobility data. Model performance is thus a guide for the user as to the degree of predictability in the predictors they have chosen with respect to the class that they have chosen to predict. A rigorous analysis of model performance can be found in reference [47]. We have added some relevant text summarizing these points. |
| The abstract is overly brief and lacks sufficient details. Expanding it more on the aspect of methodology and implication is recommended. | It was edited and expanded |
| The abstract needs to be revised and edited for improved clarity and simplicity. | Abstract was edited and expanded. |
| Conceptual and Computational Frameworks: The paragraphs under both topics are very elaborative and verbose (some are more than 20 lines), but it is overwhelming and makes it challenging to follow the flow of ideas. So, breaking it into smaller paragraphs and making it precise will enhance clarity and better understanding. | We have revised the two sections and rationalized both, eliminating unnecessary text and making them more readable. |
| - Some of the references have a reference code and most don’t have it. For example, reference number 8, 23, 26, 30, 35, and 37. But most of the reference lacks it. So, make it uniform and correct it all according to the journal’s referencing style. | Corrected, and updated using the journal style. |
| **Reviewer 2** |  |
| The introduction part seems too short to describe some motivational facts behind this study. I would like to encourage the authors to make a robust discussion highlighting the influence of individual decision-making in uncertain environments. In addition, they may cite some articles showcasing the necessity of classical game models to trace out the opinion dynamics as well as its impact on public decision-making. Inline to that they may take help from the following textbooks/articles:  (i) Heidecke et al.; A mathematical model to assess the effectiveness of test-trace-isolate-and-quarantine under limited capacities, PLOS ONE, 2024.  (ii) Tanimoto Jun; Sociophysics Approach to Epidemics, Springer, 2021.  (iii) Kim et al.; Tracing and testing multiple generations of contacts to COVID-19 cases: cost-benefit trade-offs, ROYAL SOCIETY OPEN SCIENCE, 2022.  (iv) Tanimoto Jun; Fundamentals of Evolutionary Game Theory and its Applications, Springer, 2015.  (v) Maria Martcheva; An Introduction to Mathematical Epidemiology; Springer, 2015.  (vi) Ghosh et al.; A mathematical model for COVID-19 considering waning immunity, vaccination and control measures, Scientific reports, 2023.  (vii) Daems et al.; The Race for COVID-19 Vaccines: Accelerating Innovation, Fair Allocation and Distribution, Vaccines, 2022 | Thanks; we have added a paragraph on the relevance of modelling behavior, including one of the suggested references.  We are in full agreement about the influence of individual decision making in uncertain environments. Indeed, we have already emphasized this fact in the section describing our Conceptual Framework.  We have added text emphasizing the role of game theory as being a framework in which individual decisions can be modelled. As a theoretical model approach this is complementary to our data-driven framework, as empirical data on a large set of individual decisions in space and time does not exist. We have also added a reference Nagkoulis, N. Game-theoretical perspectives on COVID-19 pandemic. *Discov Public Health*21, 131 (2024). https://doi.org/10.1186/s12982-024-00242-6) that discusses 60 articles associated with game-theoretic approaches to COVD19. |
| In your proposed model, you have mentioned the basic division of variables fall into two categories, namely ecological and epidemiological. Can you please justify which type of variable is more influential while modeling an epidemic like COVID-19? | This is discussed in the section **Ecological versus epidemiological models**, explaining that it depends in the relevant question (who or where) as well on the available data. Thus, population density might be very relevant for predicting those municipalities with the highest infection rate (an ecological question) but not very important for predicting which residents of the municipality are likely to be infected (an epidemiological question). |
| The authors are encouraged to add different types of decision-making protocols into the probabilistic model they used. I believe, it would be more interesting for the general audience of PLOS ONE. For example, they can cite the following recently published articles to catch things up while elaborating the updating protocols.  (i). Mansura et. al.; An in-silico game theoretic approach for health intervention efficacy assessment, Healthcare Analytics, Vol. 5, 100318, 2024.  (ii). Kulsum et. al.; Modeling and investigating the dilemma of early and delayed vaccination driven by the dynamics of imitation and aspiration, Chaos, Solitons and Fractals, Vol. 178, 114364, 2024.  (iii). Alam et al.; A Game-Theoretic Modeling Approach to Comprehend the Advantage of Dynamic Health Interventions in Limiting the Transmission of Multi-Strain Epidemics, Journal of Applied Mathematics and Physics 10 (12), 3700-3748, 2022. | We think it is important to emphasize that our models are completely data-based, where we predict an important epidemiological characteristic of an epidemic using only data. The only model assumptions that enter are: which set of predictors, X, and which algorithm to use to calculate P(C\|X). This is very different to a game theoretic approach where many assumptions must be made, not least of which is which strategies to include. |
| It may also improve their current MS if the figure captions would be made more self-contained. More precisely, one could also consider a sentence or two saying what is the central theme or message of each figure. Also, the authors are requested to add more visuals that can convey the central theme of the proposed model for general readers.  In this regard, the authors may cite the following works:  (i). Tatsukawa et. al.; Stochasticity of disease spreading derived from the microscopic simulation approach for various physical contact networks, Applied Mathematics and Computation, 431(80):127328, 2022.  (ii). Alam et al.; A Game-Theoretic Modeling Approach to Comprehend the Advantage of Dynamic Health Interventions in Limiting the Transmission of Multi-Strain Epidemics, Journal of Applied Mathematics and Physics 10 (12), 3700-3748, 2022. | We have extended the figure captions to be more self-contained, as suggested.  We have also added a visual that shows our approach in diagrammatic form. |
| I would suggest the authors to extend their abstract more with the key results. As it is, the abstract is a little thin and does not quite convey the interesting results that follow in the main paper. | Abstract was updated. |
| Editor |  |
| 1. Please ensure that your manuscript meets PLOS ONE's style requirements, including those for file naming. |  |
| 2. Please note that PLOS ONE has specific guidelines on code sharing for submissions in which author-generated code underpins the findings in the manuscript. In these cases, we expect all author-generated code to be made available without restrictions upon publication of the work. | The code for EpI-PUMA is available in the following gits <https://github.com/chilam-lab/epipuma1.front.git> y <https://bitbucket.org/conabio_c3/epi-species-middleware.git> |
| 3. We note that the grant information you provided in the ‘Funding Information’ and ‘Financial Disclosure’ sections do not match.  When you resubmit, please ensure that you provide the correct grant numbers for the awards you received for your study in the ‘Funding Information’ section. | This has been corrected. |
| 4. Thank you for stating the following financial disclosure:  “We are grateful for financial support from DGAPA-PAPIIT project IV100520. “  Please state what role the funders took in the study. If the funders had no role, please state: "The funders had no role in study design, data collection and analysis, decision to publish, or preparation of the manuscript."  If this statement is not correct you must amend it as needed.  Please include this amended Role of Funder statement in your cover letter; we will change the online submission form on your behalf. | We have added a statement on the role played by the funders. |
| 5. Thank you for stating the following in the Acknowledgments Section of your manuscript:  “We are grateful for financial support from DGAPA-PAPIIT project IV100520. We have also benefited from the dedicated efforts of the whole Epi-PUMA team.”  We note that you have provided funding information that is not currently declared in your Funding Statement. However, funding information should not appear in the Acknowledgments section or other areas of your manuscript. We will only publish funding information present in the Funding Statement section of the online submission form. | We have removed the funding information from the acknowledgements section and added in the funding statement. |
| 6. We note that [Figure 1] in your submission contain [map/satellite] images which may be copyrighted. All PLOS content is published under the Creative Commons Attribution License (CC BY 4.0), which means that the manuscript, images, and Supporting Information files will be freely available online, and any third party is permitted to access, download, copy, distribute, and use these materials in any way, even commercially, with proper attribution. For these reasons, we cannot publish previously copyrighted maps or satellite images created using proprietary data, such as Google software (Google Maps, Street View, and Earth). For more information, see our copyright guidelines: http://journals.plos.org/plosone/s/licenses-and-copyright.  We require you to either (1) present written permission from the copyright holder to publish these figures specifically under the CC BY 4.0 license, or (2) remove the figures from your submission: | The EpI-PUMA 1.0 platform uses Leaflet and OpenStreet maps, both of which are freely available under a creative commons licensing. We have added a statement to this effect. The map has credit to OpenStreetMaps and a hyperlink <https://www.openstreetmap.org/copyright> as well as a credit and hyperlink to Leaflet (leafletjs.org). Leaflet’s license terms can be found at: https://github.com/Leaflet/Leaflet/blob/main/LICENSE |
| 8. Please review your reference list to ensure that it is complete and correct. If you have cited papers that have been retracted, please include the rationale for doing so in the manuscript text, or remove these references and replace them with relevant current references. | References were updated; some issues were due to an incomplete update with the references manager used (EndNote). |
