# Supplementary material for: A conceptual and computational framework for modeling the complex, adaptive dynamics of epidemics: The case of the SARS-CoV-2 pandemic in Mexico
Source: PLoS One. 2025 May 8;20(5):e0323473. doi: 10.1371/journal.pone.0323473 (PMC12061415; doi:10.1371/journal.pone.0323473)
Supplement: Attachment — Submitted filename: Reviewer comment.docx [file pone.0323473.s001.docx]

**Reviewer comment**

**Manuscript Number:** PONE-D-24-43200

**Manuscript Title: A Conceptual and Computational Framework for Modeling the Complex, Adaptive Dynamics of Epidemics: the case of the SARS-CoV-2 pandemic in Mexico**

**General comments:**

The manuscript entitled “A Conceptual and Computational Framework for Modeling the Complex, Adaptive Dynamics of Epidemics: the Case of the SARS-CoV-2 Pandemic in Mexico” by Juan Pablo Gutierrez *et al.,* demonstrates the implementation of a hybrid intelligence model that combines human and artificial intelligence to improve preparedness for the health emergencies like infectious diseases pandemics using data from the COVID-19 pandemic. The topic is interesting and the manuscript is written well. However, the manuscript is not deemed fit for publication in its current status and it needs a minor revision and improvement. I have a few comments that should be addressed:

- Including a line number would have made it much easier to comment.

1. **Abstract**

- “EPI-Puma integrates data from various sources…” Define the abbreviation “EPI-Puma” when it first appears in the manuscript. The sentence also mentions integrating data from various sources but doesn’t identify which sources are used, based on what justification the data are selected and how they are incorporated.
- The abstract doesn’t provide how accurate the EPI-puma project and platform are in predicting the outcomes.
- The abstract is overly brief and lacks sufficient details. Expanding it more on the aspect of methodology and implication is recommended.
- The abstract needs to be revised and edited for improved clarity and simplicity.

2. **Introduction:** Well-crafted and clearly conveys the purpose. Nicely presented setting the stage for the rest of the content.
3. **Conceptual and Computational Frameworks:** The paragraphs under both topics are very elaborative and verbose (some are more than 20 lines), but it is overwhelming and makes it challenging to follow the flow of ideas. So, breaking it into smaller paragraphs and making it precise will enhance clarity and better understanding.
4. **Conclusions and Recommendations:**

- Should be number 5, not 4
- It is well-written

**Reference: OK,**

- Some of the references have a reference code and most don’t have it. For example, reference number 8, 23, 26, 30, 35, and 37. But most of the reference lacks it. So, make it uniform and correct it all according to the journal’s referencing style.

**I recommend this manuscript be accepted for publication after addressing these shortcomings.**

Thanks!
